# A Gaussian extension for Diffraction Enhanced Imaging

**DOI:** 10.1038/s41598-017-18367-x

**Published:** 2018-01-10

**Authors:** Fulvia Arfelli, Alberto Astolfo, Luigi Rigon, Ralf Hendrik Menk

**Affiliations:** 1 Dipartimento di Fisica, Università di Trieste, Trieste, Italy; 2 Istituto Nazionale di Fisica Nucleare Sezione di Trieste, Trieste, Italy; 3 University College London, Department of Medical Physics and Bioengineering, London, UK; 4 Elettra Sincrotrone Trieste S.C.p.A, Science Park, Trieste, Italy; 5 Department of Medical Imaging, University of Saskatchewan, Saskatoon, SK S7N 5A2 Canada

## Abstract

Unlike conventional x-ray attenuation one of the advantages of phase contrast x-ray imaging is its capability of extracting useful physical properties of the sample. In particular the possibility to obtain information from small angle scattering about unresolvable structures with sub-pixel resolution sensitivity has drawn attention for both medical and material science applications. We report on a novel algorithm for the analyzer based x-ray phase contrast imaging modality, which allows the robust separation of absorption, refraction and scattering effects from three measured x-ray images. This analytical approach is based on a simple Gaussian description of the analyzer transmission function and this method is capable of retrieving refraction and small angle scattering angles in the full angular range typical of biological samples. After a validation of the algorithm with a simulation code, which demonstrated the potential of this highly sensitive method, we have applied this theoretical framework to experimental data on a phantom and biological tissues obtained with synchrotron radiation. Owing to its extended angular acceptance range the algorithm allows precise assessment of local scattering distributions at biocompatible radiation doses, which in turn might yield a quantitative characterization tool with sufficient structural sensitivity on a submicron length scale.

## Introduction

The translation of well-known and commonly used contrast mechanism in optical imaging to similar x-ray modalities is not straightforward. Physically this is due to the fact that the index of refraction for x-rays is very close to unity. Physiologically, stagnation in x-ray imaging might be traced back to Roentgen^1^ who doubted the feasibility of x-ray lenses due to (at that time) non-observable refraction. This explains why despite of some early pioneering work^2–4^ advanced x-ray imaging methods exploiting phase contrast have been established only in the late 1990s. In the last decades different endeavors have been undertaken to translate optical imaging methods towards x-ray imaging. To mention are here bright, dark and phase contrast schemes yielding a substantial improvement in image quality particularly for low absorbing features in soft tissue imaging. Free propagation (or in-line) phase contrast^5,6^ is the simplest implementation since it requires only a certain degree of spatial coherence of the x-ray source. It has been even applied for mammography in clinical studies with synchrotron radiation^7^ and also in clinical environment using dedicated systems^8–10^. Here typically edge enhancement is achieved by tuning the sample-to-detector distance. In order to assess refraction angles in the µrad regime, scattering or dark field in general, it requires additional optical elements. It has been shown that analyzing the deviation angle of monoenergetic x-rays penetrating tissue by virtue of perfect crystal optics in analyzer based imaging (ABI)^11–13^ increases the visibility of certain diagnostic features and pathologies in biomedical imaging^14,15^. Similar information can be retrieved utilizing grating interferometers (GI) through the Talbot effect^16^ or by edge illumination (EI)^17^ as non interferometric grating technique.

These methods have been developed and extensively applied at synchrotron radiation sources. Their angular sensitivity in the order of µrad is due to the intrinsic properties of their transmission functions. This feature in combination with dedicated post-processing algorithms permits the exploitation of contrast formation mechanisms based on x-ray refraction and ultra small angle scattering (in the order of some tenth of µrad) due to multiple refraction^18–20^. Recent efforts have also paved the way to apply these techniques to conventional x-ray tubes^21–24^. Both, grating based imaging (thus GI and EI) and ABI may yield three parametric output images, which assess different physical properties namely absorption, refraction and scattering linked to dark field images, when a minimum of three input images are acquired and dedicated image processing on a pixel basis is applied^25–28^. These modalities can be extended from planar images to computed tomography (CT)^29–31^. Both refraction and scattering are associated with the local electron density. In particular the knowledge of the refraction angle allows in principle the retrieval of the phase at least in one direction. Very recently x-ray scattering has attracted attention in imaging^32–34^ and many studies are devoted to assess scattering properties of samples. Micrometer sized particulate systems are found in many biological and non-biological porous materials. In particular suspensions of micro-spheres, micro-bubbles^16,35–38^ and alveoli^39,40^ give rise to ultra small angle scattering with significant signature in x-ray dark field images. The texture of scattering patterns can provide quantitative information in such systems, which for instance can be correlated to pathologies thus providing added value in diagnostics^41^. It is noteworthy that depending on structure and the thickness of the sample the distribution of the scattering angles can exceed some tenths of µrad.

Due to its specific transmission function (or rocking curve RC) the analyzer crystal in ABI, placed between the sample and the detector, is acting as angular filter for x-rays. It is an excellent tool for highlighting scattering when it is substantially misaligned with respect to the undeviated beam. In this case the direct beam is considerably suppressed while the transmission of scattered x-rays is enhanced. A single image acquisition is sufficient to obtain qualitative and high signal to background radiographs, however, it requires multiple image acquisition schemes to assess quantitative metrics^42–45^. These methods allow quantitatively retrieving of scattering even for wide scattering distribution, but on the cost of long acquisition times and eventually high radiation doses.

Apart from multiple image acquisition modes the approximation of the RC with Taylor expansions^13,25,46^ in combination with two or three image acquisition schemes allows to extract two or three parametric images, which subsequently results in the reduction of acquisition time and delivered radiation dose. It should be noted that these ABI based methods provide quantitative information in the angular range of the validity of Taylor expansion i.e. for small refraction and scattering angles, in the order of few µrad, which are substantially smaller than the width of the RC. If the scattering angle exceeds this limit the algorithms tend to fail and saturation is observed^47^. To overcome these limitations we have developed a novel three image based analytical algorithm referred to as Gaussian Generalized Diffraction Enhanced Imaging (G^2^DEI) capable of retrieving a wider range of refraction and scattering angles (greater than some ten µrad) that occur for instance in biological samples.

## Results

### Image analysis algorithm

**Figure 1:**
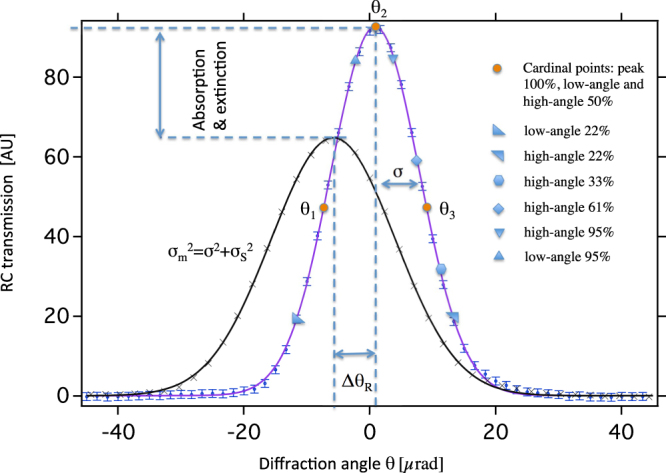
Measured rocking curves at 25 keV for a Si(1, 1, 1) analyzer in air (violet solid line is a Gauss fit on the measured data points (blue points)) and in the presents of an object featuring absorption, refraction and scattering (black solid line is the Gauss fit on the measured data points (black crosses)). Also indicated are the three cardinal points (*θ*_*i*_ with *i* = 1, 2, 3) on the two half slopes at 50% and on the peak position at 100%, where most of the simulations and experiments have been carried out. The other markers along the air-rocking curve indicate additional simulation points, which were used in the sanity check of the choice of the triad points.

The G^2^DEI algorithm proposed here traces back to the observation that an experimental transmission function of the analyzer crystal *R*(*θ*′) versus the diffraction angle *θ*′ can be approximated with reasonable fidelity by a Gaussian function with standard deviation *σ* centered around its Bragg angle *θ*_*B*_ (Fig. 1)

1 $$R(\theta ^{\prime} )={e}^{-\frac{{(\theta ^{\prime} -{\theta }_{B})}^{2}}{2\cdot {\sigma }^{2}}}\Rightarrow R(\theta )={e}^{-\frac{{(\theta )}^{2}}{2\cdot {\sigma }^{2}}}$$

Here it is assumed that the peak value of the diffraction profile of the analyzer crystal is set to unity. The simplified right hand side of equation (1) is obtained by applying the linear translation *θ*′ = *θ* + *θ*_*B*_ thus centering the RC around zero. In the presence of an object upstream the analyzer the measured RC in a detector pixel is modulated according to the local absorption, refraction and scattering properties of the sample (local RC). In the presence of absorption the amplitude of the local RC is reduced, while refraction would generate a shift of the mean value by *Δθ*_*R*_(*x*, *y*). In the presence of Gaussian scattering with standard deviation *σ*_*S*_ the measured standard deviation *σ*_*m*_ of the local RC would increase (thus *σ*_*m*_^2^ = *σ*^2^ + *σ*_*S*_^2^) while the amplitude would be reduced accordingly (extinction)^42,43^.

The statistical nature of the scattering process satisfies the introduction of a symmetric probability density function *f*(*Δθ*_*S*_, *x*, *y*)^44,45^ which is defined as a normalized Gaussian

2 $$f({\rm{\Delta }}{\theta }_{s},x,y)=\,\frac{1}{\sqrt{2\cdot \pi }\cdot {\sigma }_{S}}\cdot {e}^{-\frac{{\rm{\Delta }}{\theta }_{S}{(x,y)}^{2}}{2\cdot {\sigma }_{S}^{2}}}$$

where (*x*, *y*) describes the detection plane perpendicular to the x-ray propagation (*z*) and *Δθ*_*S*_ is the stochastic scattering angle, governed by the probability density function *f*(*Δθ*_*S*_, *x*, *y*). Consequently the intensity reaching the detector *I*(*θ*_*i*_, *x*, *y*) can be written as a angular convolution of the RC and the scattering function, thus

3 $$I({\theta }_{i})={I}_{R}\cdot {\int }_{-\infty }^{\infty }R({\theta }_{i}+{\rm{\Delta }}{\theta }_{R}+{\rm{\Delta }}{\theta }_{S})\cdot f({\rm{\Delta }}{\theta }_{S})\cdot d({\rm{\Delta }}{\theta }_{S})$$

when the analyzer is set to a given angular position *θ*_*i*_ and *I*_*R*_ is the apparent absorption. Using equations (1) and (2) and as described in the method section equation (3) can be rewritten as to

4 $$I({\theta }_{i})=\,{I}_{R}\cdot \sqrt{\frac{{\sigma }^{2}}{{\sigma }_{S}^{2}+{\sigma }^{2}}}\cdot {e}^{-\frac{{({\theta }_{i}+{\rm{\Delta }}{\theta }_{R})}^{2}}{2\cdot ({\sigma }_{S}^{2}+{\sigma }^{2})}}$$

For the ease of the discussion the peak value of the RC of the analyzer is set here to unity and the spatial coordinates are omitted. Equation (4) contains three unknowns, namely the apparent absorption *I*_*R*_, the refraction angle *Δθ*_*R*_ and the width *σ*_*S*_ of the small angle scattering distribution. Recording a minimum of three images *I*(*θ*_*i*_) = *I*_*i*_ at three different angular positions *θ*_*i*_ with *i* = 1, 2, 3 allows to solve for the unknowns, *Δθ*_*R*_, *σ*_*S*_ and *I*_*R*_:

5 $${\rm{\Delta }}{\theta }_{R}=\,\frac{{\rm{ln}}(\frac{I({\theta }_{3})}{I({\theta }_{2})})\cdot ({\theta }_{2}^{2}-{\theta }_{1}^{2})-{\rm{ln}}(\frac{I({\theta }_{1})}{I({\theta }_{2})})\cdot ({\theta }_{2}^{2}-{\theta }_{3}^{2})}{2\cdot [{\rm{ln}}(\frac{I({\theta }_{1})}{I({\theta }_{2})})\cdot ({\theta }_{2}-{\theta }_{3})-{\rm{ln}}(\frac{I({\theta }_{3})}{I({\theta }_{2})})\cdot ({\theta }_{2}-{\theta }_{1})]}$$

6 $${\sigma }_{S}^{2}=\,\frac{({\theta }_{2}-{\theta }_{1})\cdot ({\theta }_{2}-{\theta }_{3})\cdot ({\theta }_{1}-{\theta }_{3})}{2\cdot [\mathrm{ln}(\frac{I({\theta }_{1})}{I({\theta }_{2})})\cdot ({\theta }_{2}-{\theta }_{3})-\,\mathrm{ln}(\frac{I({\theta }_{3})}{I({\theta }_{2})})\cdot ({\theta }_{2}-{\theta }_{1})]}-{\sigma }^{2}$$

7 $${I}_{R}=\,I({\theta }_{i})\cdot \frac{\sqrt{{\sigma }_{S}^{2}+{\sigma }^{2}}}{\sigma }\cdot {e}^{\frac{{({\theta }_{i}+{\rm{\Delta }}{\theta }_{R})}^{2}}{2\cdot ({\sigma }_{S}^{2}+{\sigma }^{2})}}$$

The results from equations (5) and (6) can be used then by equation (7) to eventually calculate the apparent absorption image *I*_*R*_ using arbitrarily one of the three acquired images. Here the apparent absorption (equation (7)) possesses attenuation and the so-called extinction contrast^13^ i.e. the rejection of x-ray scattering, which is not transmitted by the analyzer. The refraction image (equation (5)) represents a quantitative map of the refraction angles. In other words the pixel values reflect the average deviation angle of x-rays from their original path. In contrast pixel values in the scattering or dark field image (equation (6)) represent the variance of the scattering distribution in that pixel.

It should be noted that in the absence of scattering the apparent absorption and the refraction image could be calculated from two images only. In this case equation (7) simplifies to

8 $${I}_{R}=\,I({\theta }_{i})\cdot {e}^{\frac{{({\theta }_{i}+{\rm{\Delta }}{\theta }_{R})}^{2}}{2\cdot {\sigma }^{2}}},\,\,\,i=1,2$$

and equation (5) becomes

9 $${\rm{\Delta }}{\theta }_{R}=\frac{{\sigma }^{2}}{({\theta }_{2}-{\theta }_{1})}\,\cdot \,\mathrm{ln}(\frac{I({\theta }_{1})}{I({\theta }_{2})})-\frac{({\theta }_{2}+{\theta }_{1})}{2}$$

In the absence of refraction, the apparent absorption image (equation (7)) simplifies to

10 $${I}_{R}=\,I({\theta }_{i})\cdot \frac{\sqrt{{\sigma }_{S}^{2}+{\sigma }^{2}}}{\sigma }\cdot {e}^{\frac{{({\theta }_{i})}^{2}}{2\cdot ({\sigma }_{S}^{2}+{\sigma }^{2})}},\,\,\,i=1,2$$

and the scattering image (equation (6)) to

11 $${\sigma }_{S}^{2}=\frac{({\theta }_{2}^{2}-{\theta }_{1}^{2})}{2\cdot \,\mathrm{ln}(\frac{I({\theta }_{1})}{I({\theta }_{2})})}\,-{\sigma }^{2}$$

In order to assess the capability of the new G^2^DEI algorithm it has been verified on simulated analyzer based images and has been applied experimentally to a phantom and to biological samples.

### Simulated data

**Figure 2:**
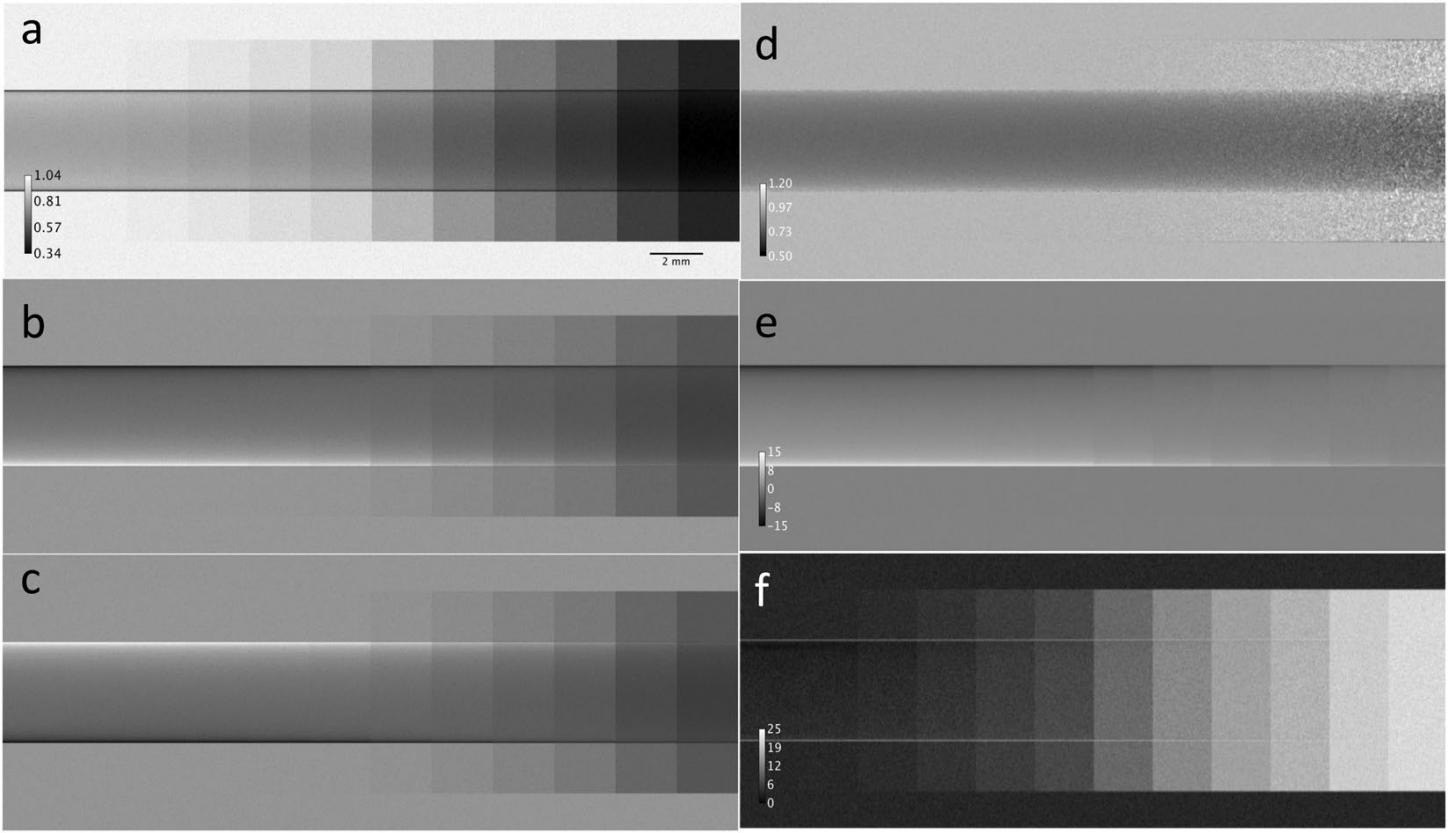
Images of the simulated phantom, (**a**) for the peak position of the analyzer, (**b**) for the low and (**c**) the high half slope. The resulting parametric images obtained applying the G^2^DEI algorithm are (**d**) the apparent absorption, (**e**) the refraction and (**f**) scattering image, respectively.

Images of the simulated phantom, which is described in the methods section, are presented in Fig. 2. In brief the simulated phantom consists of a PMMA rod, which is the source of absorption and refraction superimposed with 12 scatter foils of different thickness. The latter are characterized by different scattering distribution widths *σ*_S_ ranging from 0 to 21 µrad, which is considerably larger than the *σ* ~ 8.6 µrad of the assumed Si(1, 1, 1) RC at 17 keV. As expected, the image acquired at the peak intensity (100%) of the RC (Fig. 2(a)) is symmetric with respect to the horizontal axis of the rod and shows a clear influence of scattering for almost all paper layers, which appear as vertical bands of increasing gray levels. Already for scattering distributions with *σ*_S_ ≪ *σ* the scattered x-rays are not completely transmitted through the analyzer system since it acts as a band pass filter described mathematically by the convolution integral in equation (3). The transmission decreases progressively with the increase in *σ*_S_. For *σ*_S_ ≥ *σ* strong influence of extinction is present which can be observed at scatter foils superimposed on the right hand side of the rod.

Refraction effects are evident in the low-angle slope image (Fig. 2(b)) simulated at 50% of the peak intensity (indicated in Fig. 1). The horizontal boundaries of the rod appear lighter and darker, respectively, compared to the gray background. Obviously the opposite effect occurs in the high-angle slope image (Fig. 2(c)) simulated at 50% of the peak intensity.

The associated parametric images, apparent absorption *I*_*R*_(*x*, *y*), refraction *Δθ*_*R*_(*x*, *y*) and scattering *σ*_*S*_^2^(*x*, *y*), reconstructed according to equations (5–7) for the aforementioned triad of points (low-angle 50%, peak position, high-angle 50%), are presented in Fig. 2(d–f), respectively. In the apparent absorption image (Fig. 2(d)) only absorption effects of the PMMA rod are revealed. Interestingly the algorithm can recover extinction even at the horizontal boundaries of the rod also in the presence of wider scattering distributions. Only for the thickest scattering foil (when the standard deviation of the scatter distribution becomes 21 µrad) extinction effects seem to slightly emerge in the apparent absorption image as speckle pattern in the right hand side of the image. This spurious contamination of scattering in the apparent absorption image is due to the noise inherent in the result of equation (7) in case of large attenuation: the latter implies a reduced number of collected photons and thus large pixel-to-pixel fluctuations.

**Figure 3:**
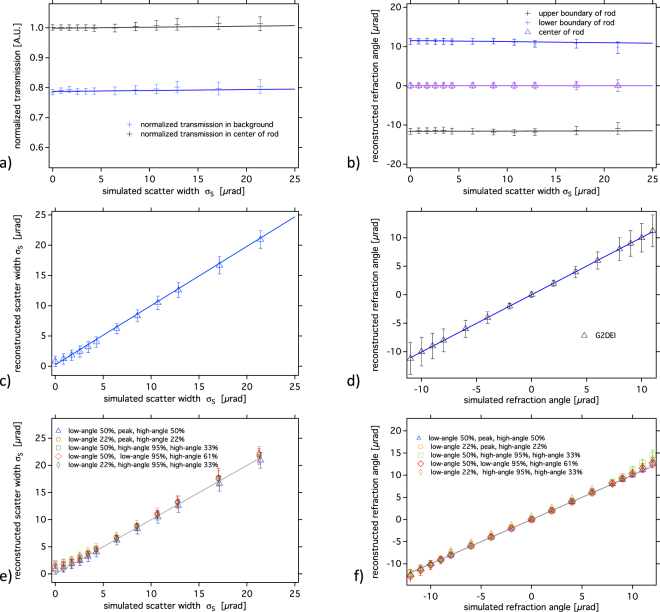
Normalized absorption profiles (**a**) in the background and in the center of the rod versus the simulated scatter width. (**b**) Reconstructed refraction angle versus the scatter width in different vertical positions in the rod, (**c**) reconstructed scattering width versus the simulated scatter width together with a line fit (solid line), (**d**) reconstructed refraction angle versus the simulated refraction angle with a line fit (solid line). (**e**) Reconstructed scattering width versus the simulated ones and (**f**) reconstructed refraction angles versus the simulated ones for different combinations of points on the RC. In both cases unity slope lines have been added.

Quantitatively this is shown by the progressively increasing error bars in Fig. 3(a), in which the reconstructed intensity within and outside the rod is reported versus the standard deviation of the simulated scatter distribution. Regardless of the latter, the measured transmission equals the theoretical one within the error bars and can be quoted with 0.79 for the highest absorption in the rod and with 1.0 outside the rod.

As expected, the refraction image (Fig. 2(e)) is quite uniform in the central part of the rod phantom and features almost the same gray value as in the background, which is null within the error bars. The reconstructed refraction angles at the horizontal boundaries of the rod are represented by white (or positive) for the leading edge and black (or negative) for the trailing edge.

The refraction angle versus the standard deviation of the scattering distribution is presented in Fig. 3(b). Reported here are the reconstructed values for the leading and trailing edge, respectively, and for the flat central part (close to 0 µrad) of the rod. For those parts of the phantom in which no scattering is superimposed the measured refraction angle is equal to the theoretical value (from −12 µrad to 12 µrad) at the boundaries of the rod and null within the error bars for the center. Within the error bars, which increase with increasing scattering width, the reconstructed refraction angle remains constant.

The scattering image (Fig. 2(f)) shows that the G^2^DEI algorithm is capable of extracting the width of scattering distribution with insignificant contamination also in the parts where strong refraction and absorption are present. The twelve simulated scatter foils are visible and distinguishable after reconstruction. For a quantitative analysis, the reconstructed scattering distribution versus the simulated scatter width is reported in Fig. 3(c). For all values the reconstruction retrieves the simulated values within the error bars.

The chart in Fig. 3(d) assesses the potential of the algorithm for reconstructing refraction angles versus the theoretical ones from −12 µrad up to 12 µrad for the rod of this phantom for a given scattering width *σ*_S_ = 6.42 µrad. It is noteworthy that in case of a rod the refraction angle will exceed 10 µrad (positive or negative) only at ~99.5% of the radius. The G^2^DEI method yields a linear dependency for the reconstruction of refraction angles from −12 µrad up to 12 µrad thus in most parts of the rod.

Noticeable scattering is also visible as single pixel wide (14 µm) bands at the horizontal edges of the rod in scatter image (Fig. 2(f)). This is actually an artifact caused by the finite detector pixel size which is also observed for instance when using the MIR technique. In the proximity of the border of the rod, the photons are deviated by progressively larger refraction angles sprawling the length scale of one pixel. Therefore, these edge pixels see a distribution of refraction angles, which is interpreted as scattering by the algorithm. Moreover, the pixels at the very edge of the rod collect a mixture of highly refracted x-rays and a part of the incident beam that does not intersect the rod. The resulting bimodal angular spectrum of this peculiar distribution leads to a large value of the computed width of the scattering distribution^42^.

In order to demonstrate the general validity of the new equations and the robustness of the algorithm we present an additional study based on simulations in which the choice of different triad of points along the RC has been assessed. The results for the reconstruction of the width of the scattering distributions and of the refraction angles, respectively, are depicted in Fig. 3(e) and (f). As in Fig. 3(c) and (d), unity slope lines have been added in the charts for reference. Within the error bars the reconstructed width of the scattering distributions in Fig. 3(e) and the reconstructed refraction angle (Fig. 3(f)) possess unity slope. For the latter a single scattering foil contributing with a constant scattering width of *σ*_S_ = 6.42 µrad was assumed. In both cases the algorithm delivers robust results even when the set of three working points is different from the typical choice using the two half slopes and the peak position.

### Experimental data

**Figure 4:**
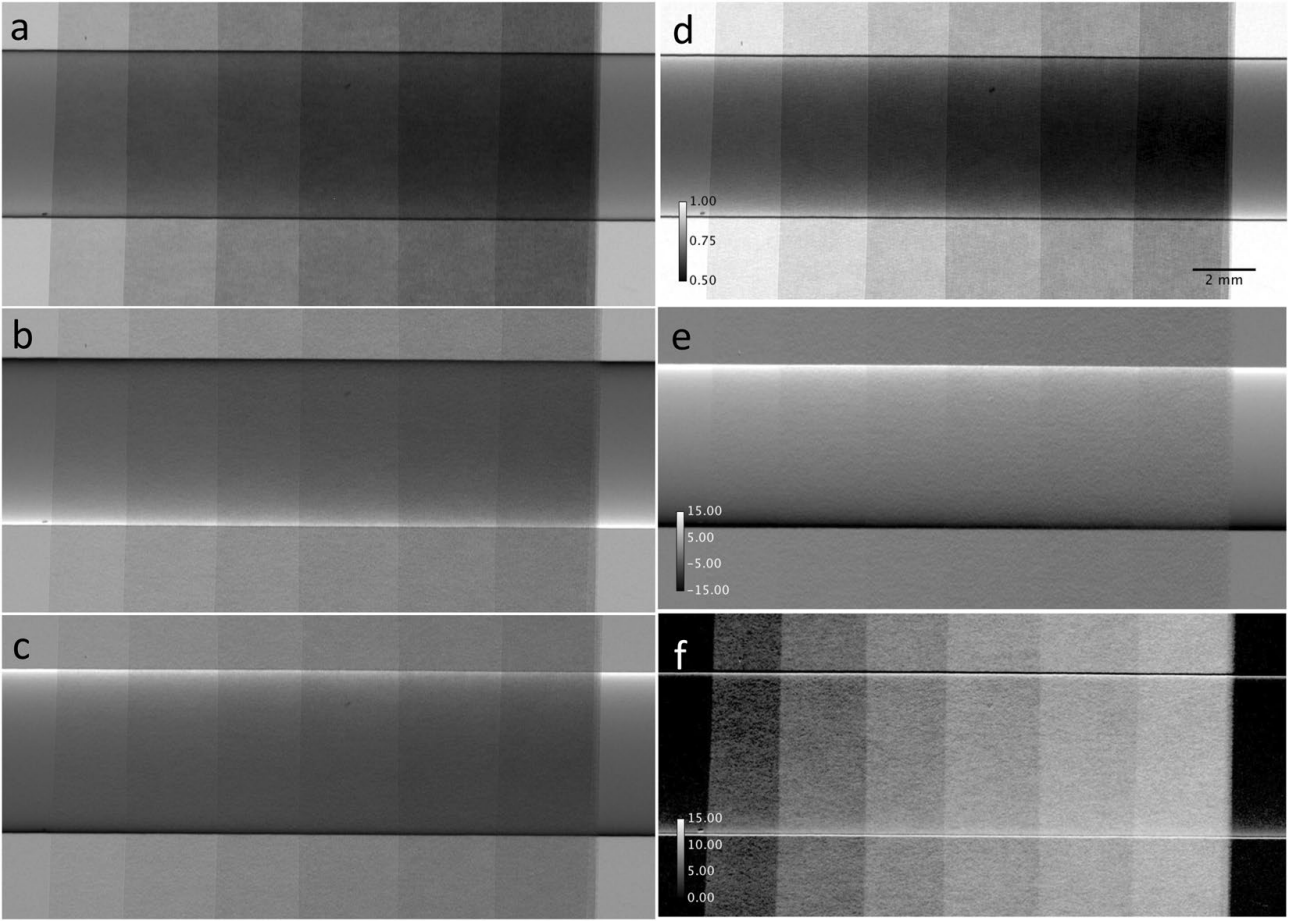
Images of the experimental data. Shown are (**a**) the peak image, (**b**) the low and (**c**) the high-angle images, respectively. The parametric images are (**d**) the apparent absorption, (**e**) the refraction and (**f**) the scattering image.

**Figure 5:**
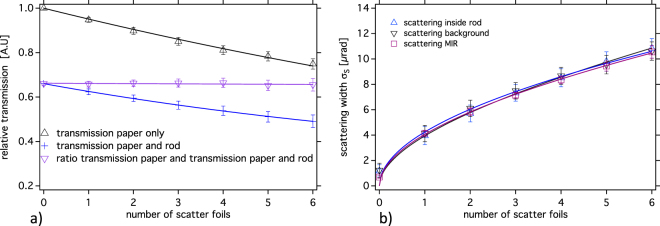
(**a**) Experimental normalized transmission profiles in the background and in the center of the rod versus the number of scatter foils. The solid lines are exponential fits on the measured data points. (**b**) Reconstructed scattering width versus the number of scatter foils (triangles) superimposed with a square root fit (solid lines). For reference the reconstructed scattering width using the MIR technique are displayed as well.

Acquired images at 17 keV photon energy of the experimental phantom, which is similar to the simulated one and is described in the method section, are presented in Fig. 4(a–c). The parametric images calculated according to equations (5–7) are reported in figures Fig. 4(d–f), respectively. The apparent absorption image (Fig. 4(d)) shows the absorption of the PMMA rod and differently from the simulations also the attenuation of x-rays by the paper, which is a composite of cellulose (C_6_H_10_O_5_) and among other additives most importantly calcium carbonate (CaCO_3_) filler^48^. The paper attenuation can be quoted with (2.7 ± 0.7) %, (6.9 ± 0.9)%, (10.9 ± 1.2) %, (14.8 ± 1.3)%, (17.9 ± 1.5)%, (21.3 ± 1.5)% for the thinnest layer to the thickest layer. In regions inside the rod and in the background the transmission follows an exponential decay with the number of scatter foils with high reliability (Fig. 5(a)). Normalizing the rod and paper transmission to the pure paper transmission indicates that the transmission through the central part of the rod remains constant at 0.66 ± 0.02 for different layer thickness as expected for its pure absorption and is consistent with the theoretical value of 0.66 for 5 mm PMMA at this energy.

Regarding the refraction image (Fig. 4(e)) the algorithm is capable of reconstructing refraction signals from −11 µrad to 11 µrad, which are the theoretical values at the two boundaries of the rod. In contrast to the simulations where the foils have been assumed as homogenous, non-absorbing and non-refracting scatterers, here the textile structure of the paper gives rise to additional multiple refraction, which is the source of speckle in the refraction image.

The scattering image (Fig. 4(f)) reveals only the presence of the scattering produced by the paper layers, while the absorption and refraction effects are mostly removed with the exception of the small bands along the boundary of the rod. As already noticed in the simulation (Fig. 2(f)) these are artifacts, which can be explained as before by the finite detector pixel size.

Within the error bars the behavior of the reconstructed scatter width is similar in regions within and outside the rod absorber (Fig. 5(b)). As expected from the statistical nature of the scattering process, the width scales with the square root of the layer thickness^44^, thus with the number of layers. An independent measurement with MIR technique^42^ using 30 images along the rocking curve, which is included in this figure as well, reveals that the G^2^DEI algorithm has the ability to reconstruct the values of the scatter width within the error bars, which are in the order of ±1.3 µrad.

### Biological samples

**Figure 6:**
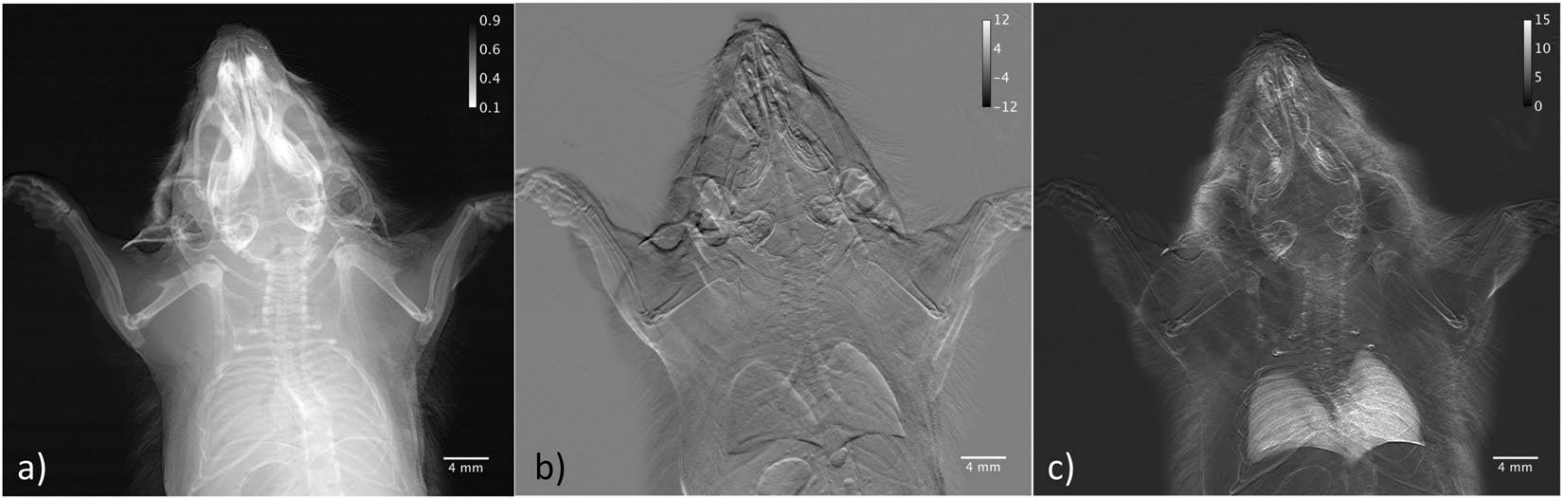
Postmortem radiographic images of a mouse, parametric images: (**a**) apparent absorption, (**b**) refraction and (**c**) scattering.

**Figure 7:**
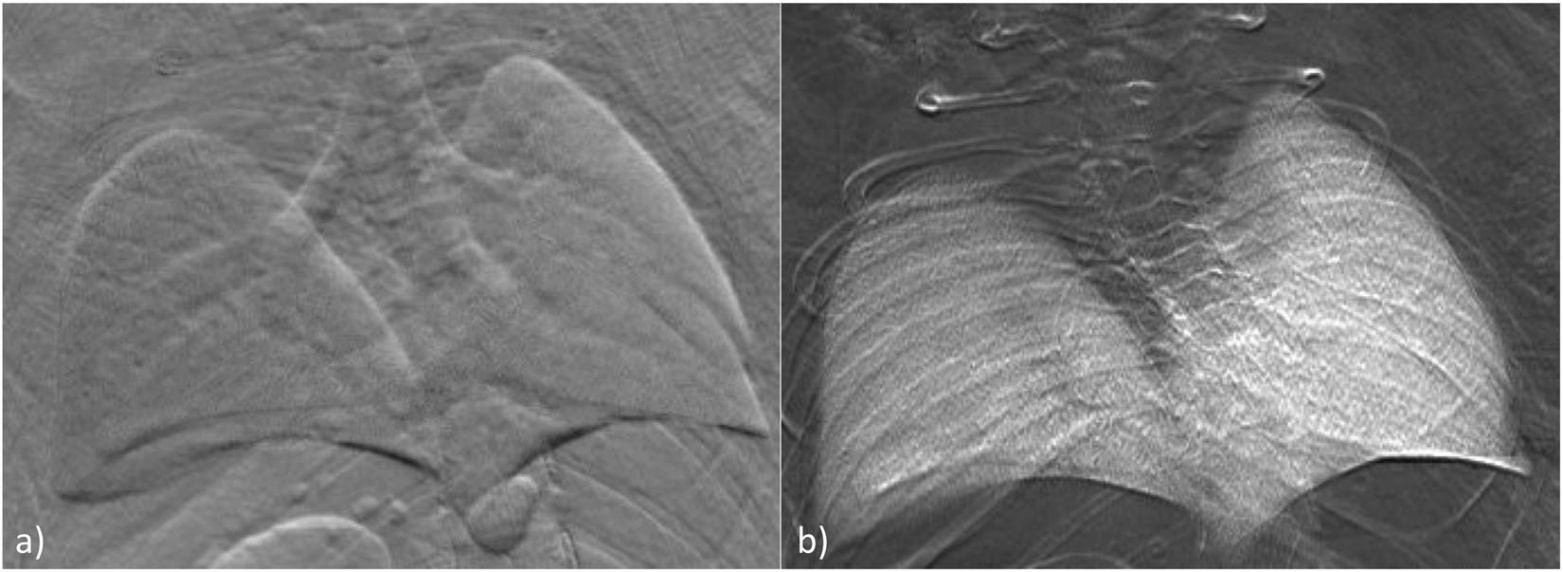
Full resolution radiographic images displaying lung details of (**a**) the refraction image and (**b**) the scattering image.

Postmortem parametric images at 25 keV photon energy of a torso of mouse are presented in Fig. 6 where attention was drawn on the lungs. The transmission in the apparent absorption image (Fig. 6(a)) ranges from 10–90%. Visible in the refraction image (Figs 6(b) and 7(a)), in which the displayed angles are ranging from −12 µrad to +12 µrad, is the trachea branching into two bronchi. The right bronchus bifurcates close to the middle and inferior lobes. Superior lobe and post-caval lobe as well as signatures from the annular cartilaginous ligaments of trachea and bronchi can be appreciated to a certain extent. The boundaries of the pleura give raise to refraction, which is in the order of −5 µrad and +5 µrad for the low-angle and high-angle side.

In the scattering image the lungs appear as areas of high intensity signal (area contrast), which is modulated by local thickness of the organ and thus by the number of alveoli intersecting the x-ray path. The scattering widths displayed in the scattering image (Figs 6(c) and 7(b)) in terms of standard deviation are ranging from 0 µrad (black) to 15 µrad (white) whereas the average in the right lobe is about (10 ± 1.5) µrad, in the upper part of the superior lobe (7.9 ± 1.0) µrad and (11.5 ± 1.1) µrad in the lower part of the inferior lobe. The average value in the left lobe is (8.8 ± 1.2) µrad.

Since the average size of the alveoli in live mammalian lung (mice) is about ∅ 60 µm^49^ and the alveolar density for adult mice is found^50^ to be 3071 ± 740/mm^3^ the x-rays will intersect in average ~15 alveoli/mm in depth keeping in mind that lungs terminate in acini, many-lobed sacs containing groupings of alveoli. When traversing 10 mm of lung tissue x-rays interact in average with 150 alveoli generating a scattering distribution, which is very similar to that from multiple particle systems, with a standard deviation of about 10 µrad^51^. Within the error bars this value matches with the standard deviations of the scatter distribution retrieved from the scatter image.

**Figure 8:**
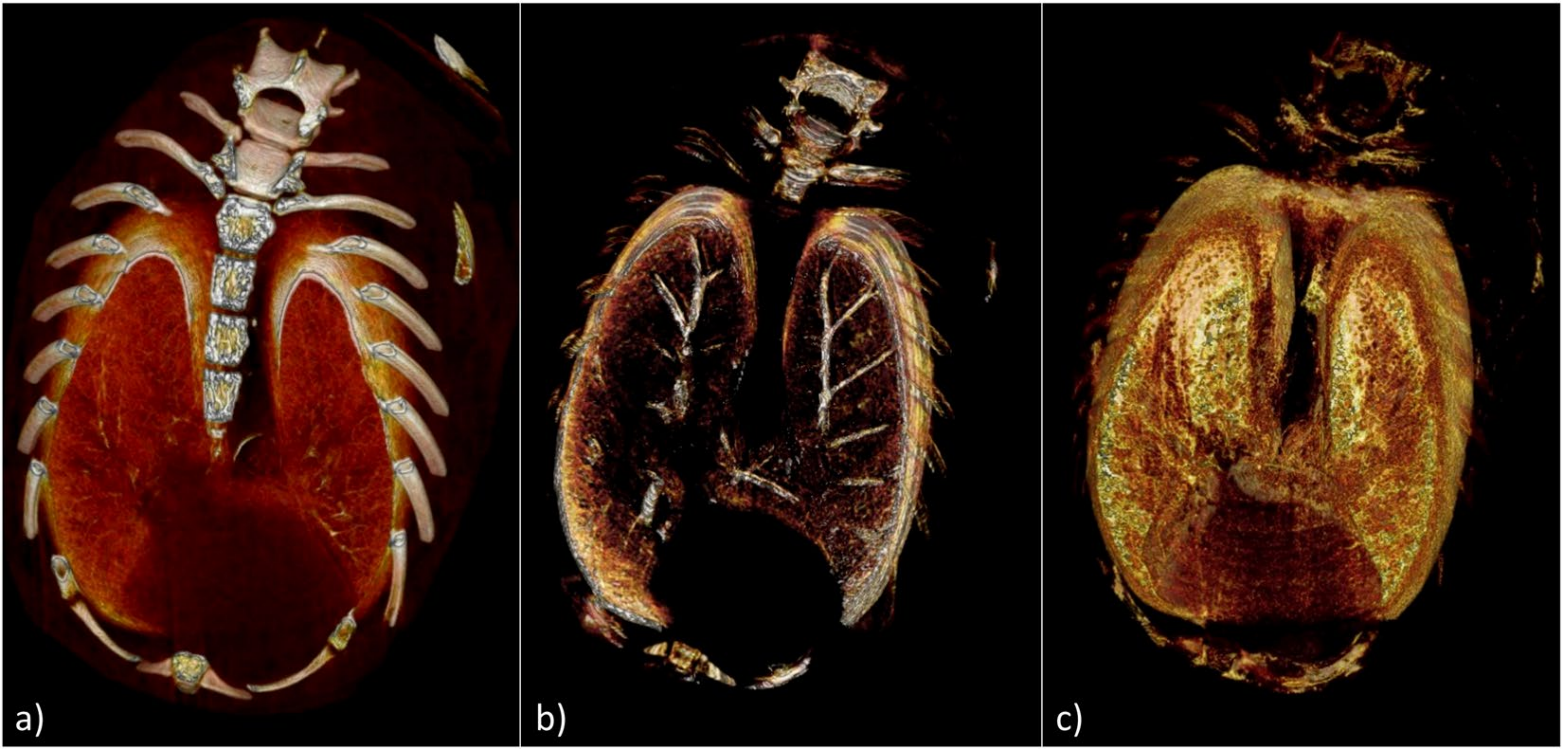
3-D CT reconstruction of the parametric data volumes of the mouse lung: (**a**) apparent absorption, (**b**) refraction and (**c**) scattering image.

The application of the G^2^DEI algorithm to volumetric data is qualitatively shown in Fig. 8 where 3-D rendering of 1 mm thick sections of parametric CT data sets are shown. As expected the apparent absorption image (Fig. 8(a)) renders bones and, due to extinction, lung tissue very well, while the refraction image (Fig. 8(b)) specifically highlights pleura and bronchi. In the scattering image (Fig. 8(c)) lung tissue is modulated according to the number of alveoli, which have been intersected by the x-rays. Absorption and refraction features such as ribs, spine and bronchi are mainly removed with the exception in the upper part of the reconstructions where the associated refraction angles exceed the linear range of the G^2^DEI algorithm.

**Figure 9:**
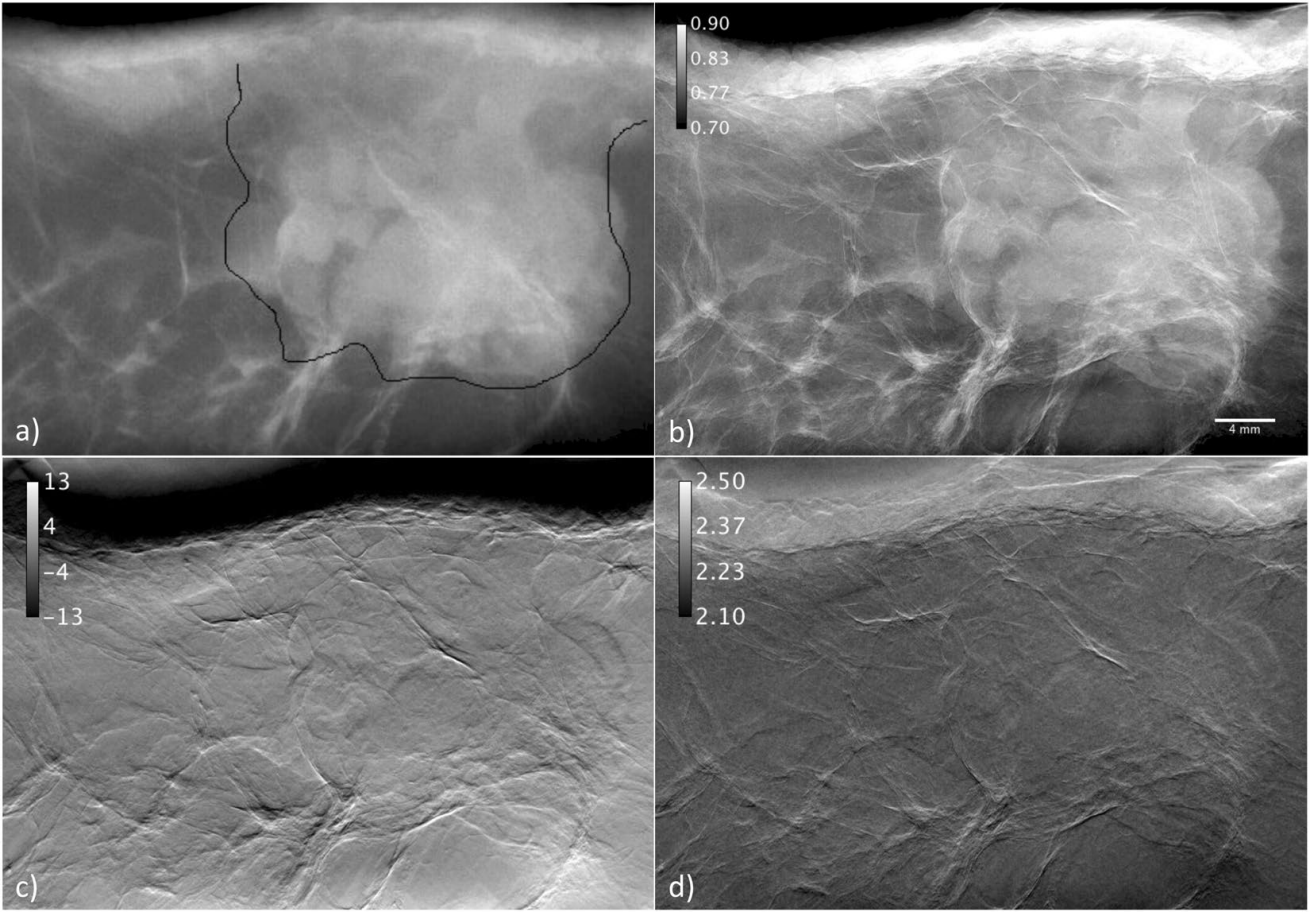
(**a**) Radiograph of a breast tumor obtained with a clinical mammographic device and (**b**) the apparent absorption image. (**c**) Refraction image and (**d**) scattering image.

The tumor bearing breast sample was a 1.5 cm thick slab of a mastectomy, which had been fixed in 4% neutral buffered formalin. The sample was approximately 3 cm high and 6 cm wide and images are shown in Fig. 9. For comparison a conventional image (Fig. 9(a)) was acquired at the digital mammography unit (Senographe DS, GE Healthcare) of the Cattinara Hospital in Trieste with x-ray tube settings of 24 kV, 63 mAs, anode Mo/Mo (anode/filter) where the free hand outline shows the tumor region which is almost completely filled by a solid-pattern, poorly differentiated carcinoma (grade 3). The infiltration of the tumor into the surrounding tissue is enhanced in the reconstructed apparent absorption image (Fig. 9(b)). The transmission here ranges from 0.71 ± 0.02 for adipose tissue to 0.59 ± 0.02 for tumor tissue. As expected both spatial and contrast resolution are substantially higher with respect to the conventional absorption radiography in Fig. 9(a). In the refraction image in Fig. 9(c) the infiltrating strands moving from the tumor towards the surrounding tissue are visible over the fat tissue. Depending on the strand size the associated (absolute) refraction angles are extending in average from approximately 3 µrad to 10 µrad, thus in the linear range of the algorithm. A band of strong refraction signals (>20 µrad) can be recognized in the upper part of Fig. 9(c), which is due to the bend of the plastic wrap, in which the tissue was imaged.

Values in terms of standard derivation in the scattering image Fig. 9(d) are ranging from 2.10 µrad (black) to 2.50 µrad (white) whereas the average in the tumor region is given by (2.23 ± 0.01) µrad while in the adipose tissue it is (2.22 ± 0.01) µrad, thus not significantly different. In the parts of the image, where the plastic wrap contributes with high absolute refraction angles a residual component of refraction of about 0.05 µrad can be appreciated in the scattering image. Due to the residual refraction at the boundary of the infiltrating strands their morphology is still visible, however the overall scattering features, thus the dark field signature between breast tissue and tumor, are insignificant in this angular regime below some tenth of µrad. In this context it is emphasized that the algorithm delivers excellent results at biocompatible radiation doses.

## Discussion

Linking the (sub) micro-structure to morphological changes in soft tissues at biocompatible radiation doses is a yet challenging problem due to the lack of quantitative characterization tools possessing sufficient structural sensitivity at this length scale. In this view dark field or scattering based images possess additional valuable information on the microscopic range without necessarily employing a high-resolution imaging detector. The latter is of interest since in direct x–ray imaging the radiation dose scales generally with the inverse square of the pixel size for details larger than the pixel. In special cases when the detail size matches the pixel size of the imaging system, for instance an alveolus which fills a detector pixel, the radiation doses scales with the inverse fourth power^52^. In general x-ray small angle scattering is observed when the microscopic correlation radius between object features is substantially smaller than the pixel size of the image receptor. With other words, one can assume that the pixel aperture in the object plane collects the whole distribution of scattering angles produced by the sample. The robust yet analytical G^2^DEI algorithm based on a Gaussian approximation of the transmission curve of the analyzer (RC) described in this manuscript enables us to qualitatively assess such scattering in a wide angular validity range.

For biological samples this in turn might yield information on biological function. Scattering can be efficiently separated from refraction and absorption effects acquiring only three images of the sample with the associated dose reduction with respect to multiple images approaches. In addition all parametric images possess high contrast and detail visibility. At the same time the G^2^DEI overcomes the limitations of alternative methods for ABI based on first and second order Taylor expansions^13,25,27^ that provide excellent results, however, only as long as the associated refraction and scattering angles are comparable to the linear Taylor approximation of the slopes of the RC.

It should be noted that the G^2^DEI algorithm delivers robust results for arbitrary chosen working points, which implies that no special symmetry is assumed *a priori*. This is advantageous for practical implementation of ABI experiments. As matter of fact, most datasets are typically collected at cardinal working points comprising the two half slopes and the peak position. However, when switching rapidly between these working points (as it is required for instance in *in-vivo* imaging) it may be difficult to match exactly the preset positions, even if the angular resolution of the set-up is sufficiently high. Quite often, slight differences between the intended and the actual working points are observed. In this case, the flexibility of the algorithm allows making up for these differences. Consequently G^2^DEI allows exploiting efficiently triads of working points that are different from the typical set of the cardinal points.

A dedicated simulation program was used to verify the G^2^DEI algorithm. It revealed the capability to efficiently and quantitatively separate absorption, refraction and scattering with high angular dynamics covering at least three standard deviations of the RC. This means it is preserving a high sensitivity in the sub µrad regime over a large angular validity range of some tenths of µrad. This verification has been underpinned by the experimental analysis on a phantom and biological samples, which confirmed its linear response in the full angular range of the latter and its capability to retrieve precise and quantitative information on absorption and scattering at biocompatible radiation doses.

Similar to Taylor approximation approaches^13,46^ in the absence of refraction or scattering two image acquisition schemes can be performed applying a simplified G^2^DEI formalism in order to retrieve absorption and scattering or absorption and refraction, respectively with a further radiation dose reduction.

The high intensity area contrast in the scattering image of the lungs is modulated by local thickness of the organ and subsequently by the number of alveoli intersecting the x-ray path. Therefore from the measured standard deviation of the scattering distribution the density of alveoli can be estimated without the need of employing a high-resolution detector capable to resolve the single alveolus.

Although ABI finds its natural application in synchrotron environment its high sensitivity makes it a valuable tool for investigation in material science and biological studies and the development of novel post-processing algorithms can add an even higher potential to the technique. Certainly the further exploitation of the G^2^DEI algorithm depends on the translation of ABI towards compact x-ray tubes, which has already proven by pioneering studies^23,24^. In this context it should be noted that ABI does not request high spatial coherence, but relies on the monochromacity of the x-ray beam, which in turn implies the availability of powerful compact sources capable of delivering sufficient flux in a small energy bandwidth. This can be obtained for instance utilizing small-spot liquid-metal-jet microfocus source^53^ or by compact laser-driven x-ray sources^54^.

In conclusion the proposed algorithm possesses the precision of the multiple image approach together with the advantages of the three-image modality and therefore G^2^DEI can be thought a valuable tool in x-ray imaging allowing precise assessment of quantities such as absorption, refraction and scattering properties.

It can be applied to other x-ray phase contrast modalities utilizing optical elements characterized by a Gaussian transmission function.

### Methods section

#### Derivation of the image analysis algorithm

Inserting the RC (equation (1)) and the symmetric probability density function $f({\rm{\Delta }}{\theta }_{s},x,y)$ (equation (2)) into the angular convolution (equation (3)) yields the intensity transmitted onto the detector *I*(*θ*_*i*_, *x*, *y*):

$$\begin{array}{c}I({\theta }_{i})={I}_{R}\cdot {\int }_{-\infty }^{\infty }R({\theta }_{i}+{\rm{\Delta }}{\theta }_{R}+{\rm{\Delta }}{\theta }_{S})\cdot f({\rm{\Delta }}{\theta }_{S})\cdot d({\rm{\Delta }}{\theta }_{S})\\ I({\theta }_{i})={I}_{R}\cdot \frac{1}{\sqrt{2\cdot \pi }\cdot {\sigma }_{S}}\cdot {\int }_{-\infty }^{\infty }{e}^{-\frac{{({\theta }_{i}+{\rm{\Delta }}{\theta }_{R}+{\rm{\Delta }}{\theta }_{S})}^{2}}{2\cdot {\sigma }^{2}}}\cdot \,{e}^{-\frac{{({\rm{\Delta }}{\theta }_{S})}^{2}}{2\cdot {{\sigma }_{S}}^{2}}}\cdot d({\rm{\Delta }}{\theta }_{S})\\ I({\theta }_{i})={I}_{R}\cdot \frac{1}{\sqrt{2\cdot \pi }\cdot {\sigma }_{S}}\cdot {e}^{-\frac{{({\theta }_{i}+{\rm{\Delta }}{\theta }_{R})}^{2}}{2\cdot {\sigma }^{2}}}\cdot {\int }_{-\infty }^{\infty }{e}^{-{\rm{\Delta }}{{\theta }_{S}}^{2}\cdot (\frac{1}{2\cdot {\sigma }^{2}}+\frac{1}{2\cdot {{\sigma }_{S}}^{2}})}\cdot {e}^{-2\cdot {\rm{\Delta }}{\theta }_{S}\cdot \frac{({\theta }_{i}+{\rm{\Delta }}{\theta }_{R})}{2\cdot {\sigma }^{2}}}\cdot d({\rm{\Delta }}{\theta }_{S})\\ I({\theta }_{i})={I}_{R}\cdot \sqrt{\frac{{\sigma }^{2}}{{\sigma }_{S}^{2}+{\sigma }^{2}}}\cdot {e}^{-\frac{{({\theta }_{i}+{\rm{\Delta }}{\theta }_{R})}^{2}}{2\cdot {\sigma }^{2}}\cdot [1-\frac{{\sigma }_{S}^{2}}{{\sigma }_{S}^{2}+{\sigma }^{2}}]}\\ I({\theta }_{i})={I}_{R}\cdot \sqrt{\frac{{\sigma }^{2}}{{\sigma }_{S}^{2}+{\sigma }^{2}}}\cdot {e}^{-\frac{{({\theta }_{i}+{\rm{\Delta }}{\theta }_{R})}^{2}}{2\cdot ({\sigma }_{S}^{2}+{\sigma }^{2})}}\end{array}$$

when the analyzer crystal is a set at an angular position *θ*_*i*_ and all three initially unknown effects (thus apparent absorption (*I*_*R*_), refraction angle (*Δθ*_*R*_) and scattering width (*σ*_*S*_^2^)) are present. As mentioned before for the ease of the discussion the peak value of the RC of the analyzer is set here to unity and the spatial coordinates have been omitted. Recording a minimum of three images *I*(*θ*_*i*_) at three different angular positions *θ*_*i*_ with *i* = 1, 2, 3 allows to solve for the aforementioned unknown effects. The logarithm of the ratio of two appropriate pairs of images *I*(*θ*_*i*_) yields then a system of two equations of the form

$$\mathrm{ln}(\frac{I({\theta }_{1})}{I({\theta }_{2})})=\frac{{({\theta }_{2}+{\rm{\Delta }}{\theta }_{R})}^{2}-{({\theta }_{1}+{\rm{\Delta }}{\theta }_{R})}^{2}}{2\cdot ({\sigma }_{S}^{2}+{\sigma }^{2})}\,\,\,{\rm{and}}\,\,\,\,\mathrm{ln}(\frac{I({\theta }_{3})}{I({\theta }_{2})})=\frac{{({\theta }_{2}+{\rm{\Delta }}{\theta }_{R})}^{2}-{({\theta }_{3}+{\rm{\Delta }}{\theta }_{R})}^{2}}{2\cdot ({\sigma }_{S}^{2}+{\sigma }^{2})}$$

which allows to solve for *Δθ*_*R*_ and *σ*_*S*_^2^ and which are summarized in equations (5) and (6), respectively.

#### X-ray imaging Equipment and Settings

All measurements were carried out at the SYRMEP bending magnet beamline at the ELETTRA synchrotron light source in Trieste (Italy)^55^. The optics of the SYRMEP beamline features a double crystal Si(1, 1, 1) monochromator, utilized in the symmetric Bragg configuration. The monochromator spans an energy range 8.5–35 keV and is placed in vacuum. Sample, analyzer crystal and detector are located in air in the experimental hutch downstream of the monochromator at 23 m, 23.5 m and 24 m, respectively. At the sample position the maximum available cross section of the monochromatic, laminar x-ray beam is 150 mm in width and about 4 mm (FWHM) in height, which can be reduced by means of two micrometric slit systems upstream the sample. Sample and detector are placed on micrometric stages, allowing simultaneous vertical scanning through the beam and thus the acquisition of two-dimensional planar images. In addition a rotational stage allows the rotation of the sample for acquiring tomographic data sets.

The analyzer comprising of two perfect Si(1, 1, 1) crystals can be rotated around the Bragg angle *θ*_*B*_ with a nominal precision of 0.15 µrad utilizing a piezo drive^56,57^. The analyzer stage is fixed onto an optical table and mechanically decoupled from the other movement stages minimizing mechanical vibrations.

For the planar phantom images a CCD camera (Photonic Science Ltd, Robertsbridge, UK) with a field of view 28.6 × 28.6 mm^2^ has been used as detector featuring a minimum pixel size of 14 × 14 µm^2^ and which was equipped with an optical fiber taper and a 20 µm thick Gd_2_O_2_S:Tb (Gadox) screen. The pixel depth was 16 bit. The integration time was depending from the scan velocity of the sample and the detector through the stationary synchrotron beam and was in the order of some seconds. The biological samples were recorded utilizing an ARGUS CCD detector, which features a columnized CsI scintillator and was provided by Teledyne DALSA (Headquarter 605 McMurray Road Waterloo, Ontario, Canada N2V 2E9). This high quantum efficiency detector is normally applied in clinical mammography, panoramic dental imaging and general radiography applications. The field of view was about 220 (h) mm × 6.9 (v) mm with and the effective pixel size of 54 µm × 54 µm (binning 2) was used during data acquisition. For planar images the ARGUS CCD was operated in time delay integration mode. Typically the line rate was set to 200 lines/sec, the scan range was 100 mm and the scan velocity was 10 mm/sec. For tomographic data acquisition the ARGUS CCD detector was operated in static frame mode with an integration time of 200 ms per projection. As before also in this case the pixel depth was 16 bit.

All acquired images have been offset and slope corrected using the IDL package (Harris Geospatial solutions, Exelis Visual Information Solutions 385 Interlocken Crescent, Suite 300 Broomfield, CO 80021, USA) before applying the G^2^DEI algorithm. The parametric images have been retrieved using a procedure written in this IDL software, which applies the algorithm. The IDL package and the Fiji^58^ package were used for image processing and image analysis. Slice data were analyzed, manipulated and rendered with the open source Osirix software^59^ (Pixmeo SARL, 266 Rue de Bernex, CH-1233 Bernex, Switzerland).

#### Simulation

The G^2^DEI algorithm has been verified on simulated analyzer based images produced by means of a Monte Carlo code^47^ using the IDL package. The simulated object comprises of a horizontal PMMA rod (∅ = 3.6 mm) proving absorption and refraction, which is superimposed by a series a scatter foils. These scatter foils, 2.3 mm wide and 7.2 mm high, are characterized by different scattering distribution with standard deviation *σ*_*S*_ ranging from 0 to 21.41 µrad (namely 0, 0.86, 1.71, 2.57, 3.43, 4.28, 6.42, 8.57, 10.71, 12.85, 17.13 and 21.41 µrad), which corresponds to a full width at half maximum (FWHM) from 0 to 50 µrad (thus 0, 2, 4, 6, 8, 10, 15, 20, 25, 30, 40 and 50 µrad FWHM). This range was chosen to study the influence of this parameter in the applicability of the algorithm covering scattering angles much smaller, comparable and significantly larger than the *σ* of a Si(1, 1, 1) analyzer RC (~8.6 µrad) at 17 keV, which is the configuration utilized for both the simulation and experiments on the phantom. The simulated images are thought to be acquired at the low-angle and high-angle side at 50% of the maximum intensity (*θ*_1_ and *θ*_3_) and at the peak of the RC (*θ*_2_). Eventually, the algorithm presented above has been applied, thus producing the apparent absorption, the refraction and the scattering image (equations (5), (6) and (7)). In addition for the study on the influence of the choice of the triad of working points images have been simulated also at low-angle 22%, low-angle 95%, high-angle 95%, high-angle 61%, high-angle 33% and high-angle 22%.

The principle of the simulation can be briefly summarized as follows: after the definition of the object an x-ray beam is randomly generated assuming that all photons are parallel which holds in first approximation for synchrotron radiation sources. The vertical size of the field of view is slightly larger than the object. In a second step, each diced photon is supposed to transverse a uniform layer of scattering material. A refraction angle is generated according to a probability density function *f*(*Δθs*, *x*, *y*), which is assumed Gaussian and characterized by a given standard deviation *σ*_*S*_(*x*, *y*). In the third step the diced photon traverses the absorptive– refractive structure. Here the photon has a certain probability to be absorbed, otherwise the refraction angle is evaluated according to the geometry of the structure and on the basis of geometrical optics, i.e. Snell’s law. Then, the interaction of each photon with the analyzer set to a predefined angle *θ* is evaluated. As a consequence, an event can be accepted (photon reflected onto the detector) or rejected with a certain probability which is given by the value *R*(*θ* + *Δθ*_*R*_ + *Δθ*_*S*_) of the RC corresponding to the sum of refraction and scattering angles previously associated with the photon under consideration. Eventually, the finite size of the source and the spatial resolution of the detection system can be taken into account by means of a convolution of the generated signal with the (rescaled) estimated source and detector point spread functions. The intrinsic resolution is 1 µm^2^.

#### Experimental phantoms

**Figure 10:**
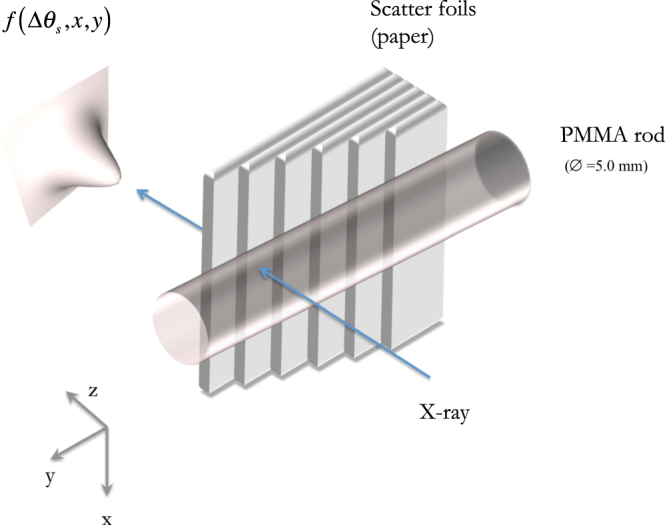
Sketch of the paper/rod phantom.

The phantom used in the experimental studies was very similar to the simulated one and is depicted in Fig. 10. It comprised two superimposed parts: (i) a PMMA rod (∅ = 5.0 mm) and (ii) a paper stairway made of up to 6 paper steps/layers (80 g/m^2^) thus covering a thickness range from 100 µm to 600 µm. Intentionally the far left and right hand side of the phantom was left blank for normalization purpose. Three images were acquired on the peak of the RC, at the low-angle side at 49% and on the high-angle side at 55% of the peak intensity at 17 keV.

#### Biological samples

Owing to it high quantum efficiency the biological tissues were acquired at 25 keV with the ARGUS CCD detector.

The first biological sample was a fresh unfixed *ex-vivo* adult CD1 mouse, which was provided by the local University animal facility. The animal experiments were carried out in accordance with the Italian Council for Animal Care guidelines for animal trials, and the Animal Care Committee of the University of Trieste approved the study. The untreated mouse was sacrificed with an overdose of CO_2_ and imaged 30 min postmortem. For the planar images the mouse was secured onto a PMMA slab, which absorbed approximately 10% of the incident x-ray intensity. Mouse images were recorded with the analyzer set on the peak position of the RC and on the two half slopes at 52% of the peak intensity at 25 keV. In this case the entrance dose was of about 0.8 mGy for each image.

For computed tomography acquisitions the same fresh animal was fixed in a Falcon tube and 900 equiangular projections over 180° have been acquired for each of the three images, which were recorded on the peak of the RC, at the low-angle side at 50% and on the high-angle side at 49% of the peak intensity at 25 keV. The entrance was about 0.6 mGy per projection. The offset and slope corrected projections have been reconstructed by a custom CT reconstruction program^60^. The parametric images have been obtained by applying the G^2^DEI algorithm on the reconstructed slice data.

The second biological sample was 1.5 cm slab of mastectomy approximately 3 cm high and 6 cm wide, which has been fixed in 4% neutral buffered formalin. The sample was prepared from a specimen of quadrantectomy and obtained from the Pathology Unit of Cattinara University Hospital of Trieste (Italy). It was handled according to local guidelines for histological examination and the work reported here was carried out following the Directive 2004/23/EC of the European Parliament and of the Council of 31 March 2004 on setting standards of quality and safety for the donation, procurement, testing, processing, preservation, storage and distribution of human tissues. For imaging the breast tissue was vacuum-sealed in a structured plastic bag and fixed on a PMMA slab.

Breast images were recorded on the peak of the RC, at the low-angle side at 54% and on the high-angle side at 49% of the peak intensity at 25 keV. The entrance dose was of about 0.8 mGy for each image, which in sum is comparable to a typical entrance surface dose in mammography.

### Data availability

The datasets generated during and/or analyzed during the current study are available from the corresponding author on reasonable request.
